# Comparison of the Efficacy of Entecavir and Tenofovir in Nucleos(T)ide Analogue-Experienced Chronic Hepatitis B Patients

**DOI:** 10.1371/journal.pone.0130392

**Published:** 2015-06-29

**Authors:** Eun Ju Cho, Jeong-Hoon Lee, Yuri Cho, Yun Bin Lee, Jeong-Ju Yoo, Minjong Lee, Dong Hyeon Lee, Su Jong Yu, Yoon Jun Kim, Jung-Hwan Yoon, Hyo-Suk Lee

**Affiliations:** 1 Department of Internal Medicine and Liver Research Institute, Seoul National University College of Medicine, Seoul, Korea; 2 Department of Internal Medicine, CHA Bundang Medical Center, CHA University, Seongnam, Korea; Yonsei University College of Medicine, REPUBLIC OF KOREA

## Abstract

The efficacy of entecavir (ETV) and tenofovir (TDF) for the treatment of nucleos(t)ide analogue (NA)-experienced chronic hepatitis B (CHB) patients has been little studied. Here, we compare the efficacy of both ETV and TDF in NA-experienced CHB patients without detectable genotypic resistance. This retrospective cohort study included consecutive NA-experienced patients who had neither current nor previous genotypic resistance and had received ETV or TDF for at least 6 months. Overall, 202 patients (146 patients in the ETV group and 56 in the TDF group) were analyzed. The cumulative probabilities of complete virologic suppression (CVS) at month 12 were 76.1% in the ETV group and 95.0% in the TDF group (*P*<0.001), respectively. The TDF-treated group achieved CVS more rapidly than the ETV group for both Hepatitis B e antigen (HBeAg)-negative and -positive patients (*P* = 0.006 and *<* 0.001, respectively), and for those with both low (< 2,000 IU/mL) and high (≥ 2,000 IU/mL) HBV DNA levels (*P* = 0.01 and 0.002, respectively). TDF group had an increased probability of achieving CVS (hazard ratio, 2.242; 95% confidence interval, 1.587–3.165; *P* = 0.001), after adjustment for HBV DNA level, the presence of HBeAg, and a history of CVS during prior treatment. During the treatment period, 23 patients (15.8%) in the ETV group developed virologic breakthrough, compared to none in the TDF group. The cumulative probabilities of developing virologic breakthrough and ETV-resistance at month 24 were 9.7% and 5.3%, respectively. In conclusion, TDF is preferable to ETV for achieving CVS in NA-experienced CHB patients without genotypic resistance.

## Introduction

The goal of treatment for chronic hepatitis B (CHB) is to prevent liver disease progression and improve survival by long-term suppression of hepatitis B virus (HBV) replication in a sustained manner [1–3]. For this purpose, present guidelines recommend a highly potent nucleos(t)ide analogue (NA), with a low rate of resistance, for the treatment of chronic HBV infection. However, because of differences in costs, drug availability, and reimbursement systems of each country, a substantial number of patients are treated with several different lines of low-potency NAs, including sequential monotherapies, add-on, or combination regimens. These treatment schemas may be problematic because of the selection of resistant HBV species.

Both entecavir (ETV) and tenofovir disoproxil fumarate (TDF) are highly potent antivirals with high genetic barriers. Recent studies directly comparing the two drugs and meta-analysis revealed that the efficacy and safety of both drugs are comparable in NA-naïve patients [4–6]. However, while ETV has limited efficacy in patients with lamivudine (LAM) resistance, TDF has been shown to achieve a potent antiviral response in these patients [7–9]. By contrast, although adefovir dipivoxil (ADV) resistance does not seem to adversely influence ETV effectiveness, TDF responses are less pronounced in patients with ADV resistance [7, 10]. Therefore, different strategies, according to the treatment history and the presence of resistance mutations at the time of treatment change, are necessary. However, there are no current guidelines regarding the use of antivirals in NA-experienced patients without detectable genotypic resistance, who are frequently treated as antiviral-naive in clinical practice. In this study, we compared the treatment response and durability of ETV and TDF in NA-exposed patients without prior or currently detectable genotypic resistance in real-life practice.

## Materials and Methods

### Study population

This retrospective cohort study included NA-experienced CHB patients who were treated with ETV or TDF monotherapy between 2007 and 2013 at Seoul National University Hospital (Seoul, Republic of Korea). Patients taking ETV or TDF for at least 6 months were included. Patients with the following conditions were excluded: serum HBV DNA <50 IU/mL; prior or current report of NA-resistant mutations; co-infection with hepatitis C, hepatitis D, or human immunodeficiency virus; history of liver transplantation; creatinine clearance <50 mL/min, as estimated by the Cockcroft-Gault formula; or missing baseline laboratory data. Patients were followed up every 3 to 6 months with virologic and biochemical assessments, including HBV DNA levels, serum alanine aminotransferase (ALT), and creatinine. The visit window was ±2 weeks. Genotypic analysis was performed at baseline and in cases of virologic breakthrough (increase of HBV DNA levels >1 log IU/mL from the nadir) or incomplete response (detectable HBV DNA after at least 6 months of therapy) during the treatment period [1, 3]. In total, 361 NA-experienced patients treated with ETV or TDF for at least 6 months were identified. One hundred fifty-nine patients did not fulfill the inclusion criteria and were excluded from analysis: 95 patients had a baseline HBV DNA of less than 50 IU/mL, 32 patients had NA-resistant mutations, 11 patients underwent liver transplantation, eight patients were co-infected with HCV, seven patients had chronic renal failure, five patients had missing baseline laboratory data, and one patient was lost to follow-up before the first visit. The remaining 202 patients were eligible for this analysis.

The study protocol conformed to the ethical guidelines of the World Medical Association Declaration of Helsinki and was approved by the Institutional Review Board of Seoul National University Hospital. Written informed consent was not required because patients received routine clinical care, and there were no additional study-specific interventions. Patient records/information was anonymized and de-identified prior to analysis.

### Study endpoints and measurements

The primary endpoint was complete virologic suppression (CVS), defined as an HBV DNA level <20 IU/mL, determined by quantitative polymerase chain reaction (PCR) assay. Secondary endpoints were a reduction in serum HBV DNA levels from baseline, normalization of serum ALT, and virologic breakthrough during the treatment period.

Serum HBV DNA levels were measured using either Abbott m2000 (lower limit of detection 15 IU/mL; Abbott Diagnostics, Chicago, IL) or Roche COBAS TaqMan (lower limit of detection 20 IU/mL; Roche Molecular System, Branchburg, NJ) quantitative PCR instruments [11]. The presence of HBV polymerase gene mutations conferring resistance to LAM (rtM204V/I/S, rtL180M), ADV (rtA181T/V, rtN236T), and ETV (rtL180M + rtM204V/I ± rtI169T ± rtV173L ± rtM250V/I/L/M ± rtT184S/A/I/L/G/C/M ± rtS202I/G) was assessed by direct PCR-based DNA sequencing [12].

### Statistical analyses

The Mann-Whitney *U* test was used to analyze differences between treatment groups, while *χ* ^*2*^ test or Fisher’s exact test was used for categorical data. A generalized estimating equations was used to assess differences in the proportion of achieving ALT normalization between treatment groups over time [13]. Times to events were estimated using the Kaplan–Meier method and compared using log-rank tests. Cox proportional hazard regression analysis was performed to evaluate independent factors for CVS or virologic breakthrough. Firth’s correction was applied in the case of a total separation of events and groups [14]. Variables with *P*<0.1 in the univariate Cox regression analysis were subjected to multivariate analysis using stepwise selection. Inverse probability of treatment weighting (IPTW), based on propensity scores, was applied to adjust for differences in baseline characteristics between treatment groups, and weighted Cox models were fitted [15]. Propensity scores were calculated using a logistic regression model based on baseline characteristics. Differences at *P*<0.05 were considered statistically significant. The analyses were performed using PASW software version 20.0 (IBM, Chicago, IL) and the R statistical programming environment, Version 3.0.2 (http://www.r-project.org).

## Results

### Study population

Of a total of 202 consecutive patients, 146 were treated with ETV (0.5 mg or 1.0 mg, once daily), and 56 patients were treated with TDF monotherapy (300 mg, once daily). The baseline characteristics of the study population are summarized in Table 1. Patients in both groups had similar baseline characteristics, except that patients in the TDF group had lower baseline HBV DNA levels (*P* = 0.02), experienced longer duration (*P*<0.001), and had multiple lines of prior treatment (*P* = 0.003) than those in the ETV group, because TDF was approved later than ETV for the treatment of HBV in Korea. S1 Table summarizes the prior NA treatment regimens. All the included patients had previously experienced one or more L-nucleoside analogue(s) (LAM, LdT or clevudine [CLV]), and most of them had received nucleoside analogue monotherapy (167 of 202 patients, 82.7%) before initiating ETV or TDF. Twelve patients (5.9%) received sequential or combination therapy with LAM and ADV, and 4 patients (2.0%) received sequential therapy with CLV and ADV. The proportion of ADV-experienced patients was not significantly different between the two groups (*P* = 0.39).

**Table 1:** Baseline characteristics by treatment group.

	ETV group (n = 146)	TDF group (n = 56)	*P*
Age (years)	52 (43–60)	48 (40–55)	0.06
Male, n (%)	83 (56.8)	27 (48.2)	0.28
Baseline serum HBV DNA (log_10_ IU/mL)	6.5 (4.4–7.6)	4.9 (3.1–6.7)	0.02
Baseline serum ALT (IU/L)	80 (40–165)	67 (29–144)	0.22
Baseline serum creatinine (mg/dL)	0.9 (0.8–1.1)	0.8 (0.6–1.0)	0.13
HBeAg–positive, n (%)	54 (37.0)	26 (46.4)	0.26
Presence of cirrhosis, n (%)	77 (52.7)	22 (39.3)	0.12
Lines of prior treatment*	1 (1–3)	1 (1–5)	0.003
Duration of prior treatment (years)	1.0 (0.6–1.6)	1.9 (1.2–3.2)	< 0.001
CVS during prior treatment	49 (33.6)	12 (21.4)	0.12
Prior treatment with ADV, n (%)	10 (6.8)	6 (10.7)	0.39

Unless otherwise indicated, data are medians, and data in parentheses are interquartile ranges.

*Data are medians, and data in parentheses are ranges.

^†^Liver cirrhosis was diagnosed when the platelet count was below 100,000/mm3 and associated splenomegaly or esophageal-gastric varices were detected.

ETV, entecavir; TDF, tenofovir disoproxil fumarate; HBV, hepatitis B virus; ALT, alanine aminotransferase; HBeAg, hepatitis B e antigen; CVS, complete virologic suppression; ADV, adefovir dipivoxil.

### Treatment responses

**Fig 1:**
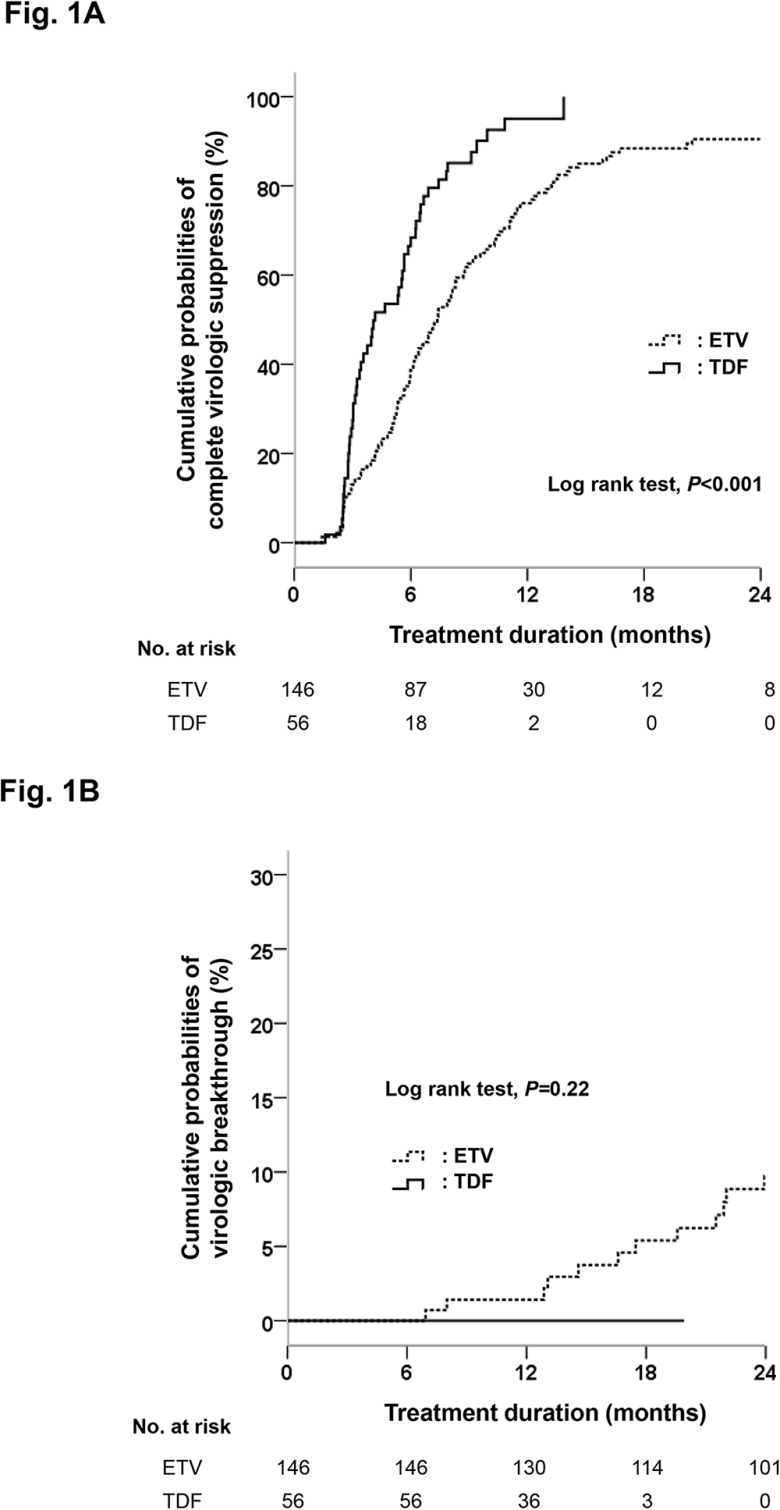
Cumulative probabilities of complete virologic suppression and virologic breakthrough by treatment group. (A) Cumulative probabilities of complete virologic suppression (defined as HBV DNA level <20 IU/mL) during the treatment period are shown for each group. (B) Cumulative probabilities of virologic breakthrough (defined as an increase in HBV DNA level >1 log10 IU/mL) during the treatment period, are shown for each group. TDF, tenofovir disoproxil fumarate; ETV, entecavir.

The median duration of treatment was 37.7 months (interquartile range, 23.4–74.5 months) in the ETV group, and 14.4 months (interquartile range, 10.6–16.3 months) in the TDF group. The TDF group showed a significantly higher probability of achieving CVS than the ETV group (*P*<0.001; Fig 1A). The cumulative probabilities of CVS at month 12 were 76.1% in the ETV group and 95.0% in the TDF group (*P*<0.001). In subgroup analysis, the TDF group achieved CVS more rapidly than the ETV group for both HBeAg-negative (median, 4.0 months vs. 6.2 months; *P* = 0.006; Figure A in S1 Fig) and-positive (median, 5.4 months vs. 9.6 months; *P*<0.001; Figure B in S1 Fig) patients. Among HBeAg-negative patients, the cumulative probability of CVS at month 12 was 85.2% in the ETV group and 95.2% in TDF group, respectively. Among HBeAg-positive patients, the rate was 60.0% and 94.9%, respectively. Similar trends were also observed for those with low (< 2,000 IU/mL) and high HBV (≥ 2,000 IU/mL) DNA levels at baseline (*P* = 0.01 and 0.002, respectively; Figure C and D in S1 Fig). In multivariate analysis, TDF treatment was significantly associated with an increased probability of achieving CVS (hazard ratio [HR], 2.242; 95% confidence interval [CI], 1.587–3.165; *P* = 0.001), after adjustment for HBV DNA level, the presence of HBeAg, and history of CVS during prior treatment (Table 2).

**Table 2:** Univariate and multivariate analyses of factors associated with complete virologic suppression.

	Univariate analysis		Multivariate analysis	
Variables	Hazard ratio (95% CI)	*P*	Hazard ratio (95% CI)	*P*
Age (per 10-year increase)	1.064 (0.931–1.217)	0.36	-	0.907
Gender (male *vs*. female)	0.988 (0.734–1.330)	0.936	-	0.645
Presence of cirrhosis	1.155 (0.858–1.554)	0.342		
HBeAg–positive	0.597 (0.437–0.816)	0.001	0.701 (0.500–0.981)	0.038
Baseline HBV DNA (log_10_ IU/mL)	0.864 (0.808–0.923)	< 0.001	0.895 (0.833–0.961)	0.002
Baseline serum ALT (IU/L)	1.000 (1.000–1.001)	0.137		
Duration of previous treatment (year)	1.068 (0.982–1.163)	0.123		
Lines of prior treatment	0.994 (0.698–1.416)	0.974		
CVS during prior treatment	1.573 (1.143–2.163)	0.005	1.469 (1.056–2.043)	0.023
Prior treatment with ADV	1.356 (0.719–2.588)	0.356		
Current regimen (TDF *vs*. ETV)	2.257 (1.608–3.165)	< 0.001	2.241 (1.588–3.164)	< 0.001

HBeAg, hepatitis B e antigen; HBV, hepatitis B virus; ALT, alanine aminotransferase; CVS, complete virologic suppression; ADV, adefovir dipivoxil; ETV, entecavir; TDF, tenofovir disoproxil fumarate.

Weighting with IPTW resulted in a good balance for baseline characteristics, including serum HBV DNA levels, duration of prior treatment, and the number of lines of prior antivirals between treatment groups (S2 Table). The TDF group achieved a higher cumulative rate of CVS than the ETV group after IPTW adjustment (*P*<0.001; S2 Fig). The cumulative probability of CVS after 12 months of treatment was 74.3% in the ETV group, and 93.8% in the TDF group, respectively. TDF treatment was independently associated with an increased probability of CVS (HR, 2.037; 95% CI, 1.372–2.959; *P*<0.001) after adjustment for other significant variables (S3 Table).

Among 145 patients with elevated ALT levels at baseline, 80.4% (82 of 102) of those in the ETV group and 74.3% (26 of 35) of those in the TDF group had normalization of ALT at month 12. The proportion of ALT normalization were comparable between the two groups overtime (*P* = 0.32, S4 Table).

### Virologic breakthrough

During the treatment period, 23 patients (15.8%) in the ETV group developed virologic breakthrough vs. none in the TDF group, although there was no statistical significance by log rank test (*P* = 0.22, Fig 1B). Among 23 patients with virologic breakthrough in the ETV group, genotypic LAM-resistance was documented in 15 patients, 13 of whom showed genotypic ETV-resistance variants. An rtA181T substitution was newly detected in one patient who received prior LAM monotherapy. The cumulative probabilities of developing virologic breakthrough and genotypic resistance to ETV were 1.4% and 0.7% at month 12, 9.7% and 5.3% at month 24, and 19.3% and 13.2% at month 48, respectively. In multivariate analysis, a history of CVS during prior treatment (HR, 0.071; 95% CI, 0.010–0.527; *P* = 0.01), and the number of lines of prior antivirals (HR, 2.628; 95% CI, 1.120–6.165; *P* = 0.03) were independently associated with virologic breakthrough in the ETV group (Table 3). After IPTW adjustment, virologic breakthrough was comparable between the treatment groups (*P* = 0.40; S3 Fig). In the weighted Cox proportional hazard model, HBeAg-positivity (*P* = 0.01), serum ALT level (*P* = 0.01), the number of lines of prior antivirals (*P*<0.001), and history of CVS during prior treatment (*P* = 0.01) were independently associated with virologic breakthrough (S5 Table).

**Table 3:** Univariate and multivariate analyses of factors associated with virologic breakthrough.

	Univariate analysis		Multivariate analysis	
Variables	Hazard ratio (95% CI)	*P*	Hazard ratio (95% CI)	*P*
Age (per 10-year increase)	1.236 (0.827–1.846)	0.301	-	0.321
Gender (male *vs*. female)	0.652 (0.286–1.487)	0.309	-	0.265
Presence of cirrhosis	0.692 (0.304–1.580)	0.382		
HBeAg–positive	2.194 (0.962–5.004)	0.062		
Baseline HBV DNA (log_10_ IU/mL)	1.112 (0.909–1.361)	0.301		
Baseline serum ALT (IU/L)	0.997 (0.993–1.001)	0.136		
Duration of previous treatment (year)	1.004 (0.722–1.395)	0.983		
Lines of prior treatment	2.598 (1.072–6.296)	0.034	2.628 (1.120–6.165)	0.026
CVS during prior treatment	0.072 (0.010–0.537)	0.01	0.071 (0.010–0.527)	0.01
Prior treatment with ADV	1.530 (0.201–11.634)	0.681		
Current regimen (TDF *vs*. ETV)	0.298 (0.002–2.584)	0.449*		

* by Firth’s correction.

HBeAg, hepatitis B e antigen; HBV, hepatitis B virus; ALT, alanine aminotransferase; CVS, complete virologic suppression; ADV, adefovir dipivoxil; ETV, entecavir; TDF, tenofovir disoproxil fumarate.

### Discussion

The present study showed that the cumulative probability of achieving CVS was higher in TDF compared to ETV monotherapy groups in nucleoside analogues-experienced patients without detectable genotypic resistance by direct sequencing. The superior efficacy of TDF over ETV in viral suppression was maintained after adjustment for potential confounding baseline characteristics using IPTW. In addition, TDF-treated patients had no virologic breakthrough during the treatment period, while ETV-treated patients showed relatively high probabilities of virologic breakthrough and ETV-resistance.

Presently, many patients have been treated with several different groups of NAs, including multiple alternating monotherapies or combination regimens. This can raise concerns in the selection of antivirals for second- or third-line therapies, because the efficacy may be compromised by prior treatment history and cross-resistance. Drug-resistant mutants selected by prior NAs are preserved in viral covalently closed circular DNA in the liver, and might even compromise the efficacy of subsequent antivirals to which HBV has never been exposed [12]. One recent study reported that prior treatment with low-potency LAM considerably affected the long-term effects of ETV, even with no evidence of genotypic resistance to LAM at baseline, and the degrees of decrease in viral titer during treatment and duration of CVS before development of resistance were both impaired [16]. In addition, LAM resistance variants were more likely to be present in LAM-experienced patients following treatment discontinuation (22%) than in LAM-naive patients (0%) [17]. These findings are supported by our present observation that within the ETV-treated group, most patients had previously received LAM monotherapy, and showed relatively high probabilities of virologic breakthrough and ETV resistance at month 48 (19.3% and 13.2%, respectively). Conversely, another study reported no difference in virologic response and development of resistance to ETV monotherapy between patients with or without prior LAM experience [9]. However, in that study, the patient follow-up period was shorter than ours (9 months vs. 39 months), and the mean HBV DNA levels were slightly lower (6.3 log_10_ IU/mL vs. 6.6 log_10_ IU/mL). Thus, it can be assumed that the duration of selection of LAM-resistant variants had influenced the treatment response and durability of ETV monotherapy.

Meanwhile, TDF monotherapy showed a potent and long-lasting response in patients with treatment failure or resistance to prior NA treatment. Neither prior exposure nor genotypic resistance to LAM affected TDF response [18]. In addition, prior ADV exposure without demonstrable resistance mutations at baseline did not significantly affect the efficacy of TDF monotherapy [7]. However, the presence of ADV resistance, especially the double mutation rtA181T/V + rtN236T at baseline, did impair TDF efficacy, although virologic breakthrough due to unique TDF resistance sequence mutations did not occur [7, 10]. Similar to these findings, we showed that the TDF group did not develop viral breakthrough and achieved CVS more rapidly than the ETV group, regardless of baseline HBeAg status or HBV DNA levels. This result is of special interest, considering that a majority of CHB patients receiving antiviral therapy may receive multiple antiviral regimens and require long-term treatment.

There are several limitations to our study. First, we used IPTW to adjust for differences between the ETV and TDF groups based on known baseline characteristics, but this retrospective, observational approach could not take into account unknown confounding. However, we tried to include all potential variables affecting treatment selection and achieve a good balance after IPTW adjustment. Second, the follow-up period of the TDF group was shorter than that of the ETV group because TDF was approved later than ETV for HBV treatment in Asia-Pacific regions (including Korea). Additional long-term studies are warranted to evaluate the efficacy and durability of TDF monotherapy in patients that have received multiple antivirals. However, no specific TDF resistance mutations have yet been identified [12]. Third, the presence of minor resistant strain(s) at baseline cannot be excluded because a specific strain can be detected by direct sequencing only if present in more than 20% of the entire quasispecies pool [19]. Classical techniques used in the clinical setting, such as direct sequencing, have limitation in the detection of variants present in very small proportions [12]. Therefore, future studies based on more sensitive method such as multiplex restriction fragment mass polymorphism or pyrosequencing may be warranted to clarify the clinical implications of the presence of low-level resistant strains. Fourth, the proportion of ADV-experienced patients was relatively small in our study because ADV was rarely used as a first-line treatment due to its limited potency with a low barrier to resistance. The superior efficacy of TDF over ETV in this study might result primarily from the prior experienced regimens mainly composed of nucleoside analogues. However, because prior ADV experience without detectable resistance mutations at baseline does not impair the efficacy of both ETV and TDF monotherapy [7, 9, 20–22], the results may be similar even though the patients had more ADV experience.

In conclusion, TDF is more effective than ETV for achieving CVS in antiviral treatment (mainly with nucleoside analogues)-experienced CHB patients. Consequently, TDF may represent a more favorable therapeutic option with regard to durability, as well as efficacy, in these patients, while careful monitoring for the development of resistance is suggested for patients on ETV monotherapy.

## Supporting Information

S1 Fig Probability of complete virologic suppression according to HBV DNA levels at baseline (Figure A and B). Probability of complete virologic suppression according to HBeAg status at baseline (Figure C and D).
(TIF)

S2 Fig Weighted probability of complete virologic suppression by treatment group. (TIF)

S3 Fig Weighted probability of virologic breakthrough by treatment group. (TIF)

S1 Table Summary of prior NA treatment regimens. (DOCX)

S2 Table Baseline characteristics by treatment group after inverse probability of treatment weighting. (DOCX)

S3 Table Univariate and multivariate analyses of factors associated with complete virological suppression after inverse probability of treatment weighting. (DOCX)

S4 Table Normalization of serum ALT. (DOCX)

S5 Table Univariate and multivariate analyses of factors associated with virological breakthrough after inverse probability of treatment weighting. (DOCX)
