# Supplementary figures and images for: Comparison of the Efficacy of Entecavir and Tenofovir in Nucleos(T)ide Analogue-Experienced Chronic Hepatitis B Patients
Source: PLoS One. 2015 Jun 29;10(6):e0130392. doi: 10.1371/journal.pone.0130392 (PMC4488001; doi:10.1371/journal.pone.0130392)

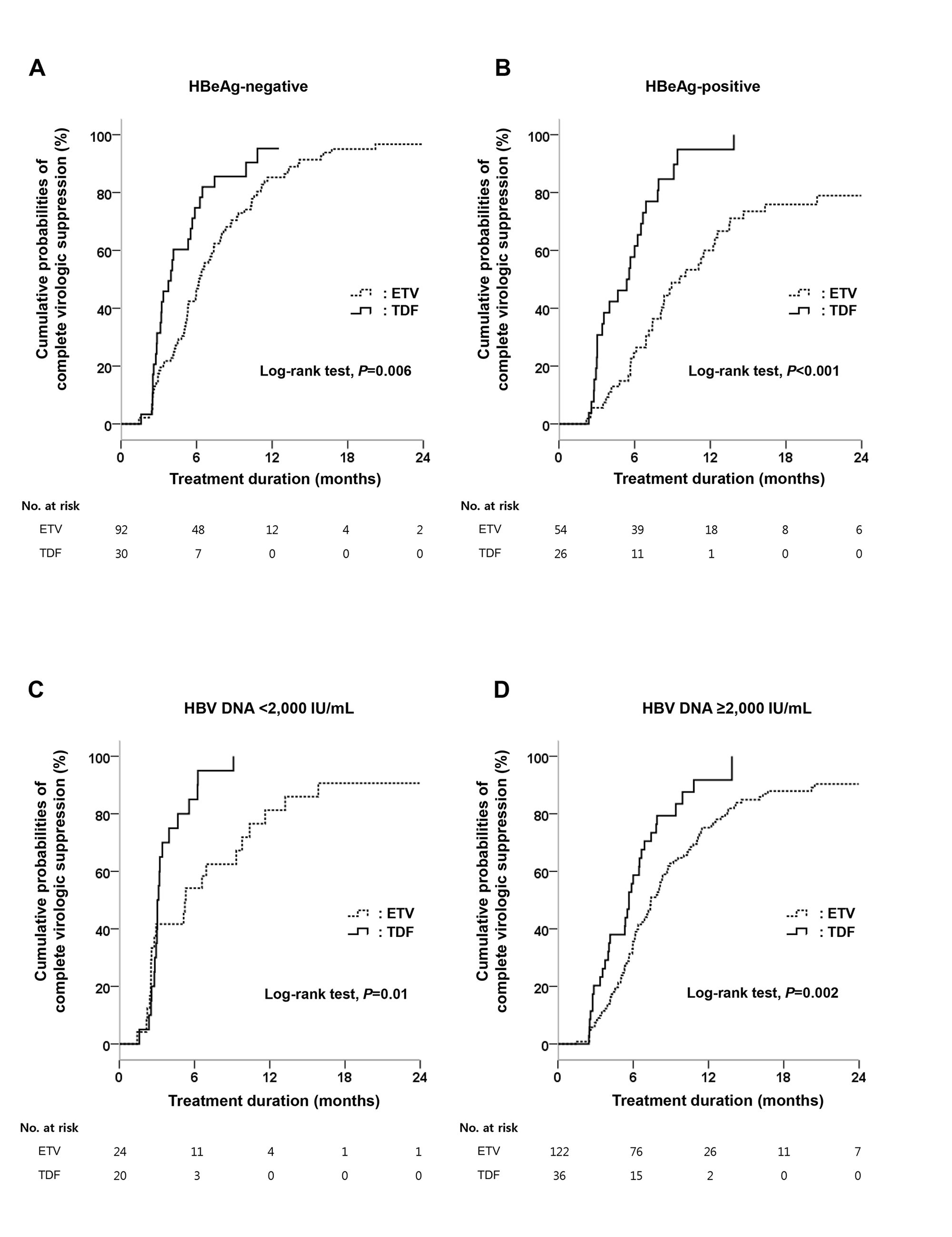

Supplement: S1 Fig — Probability of complete virologic suppression according to HBeAg status at baseline (Figure C and D). (TIF) [file pone.0130392.s001.tif]

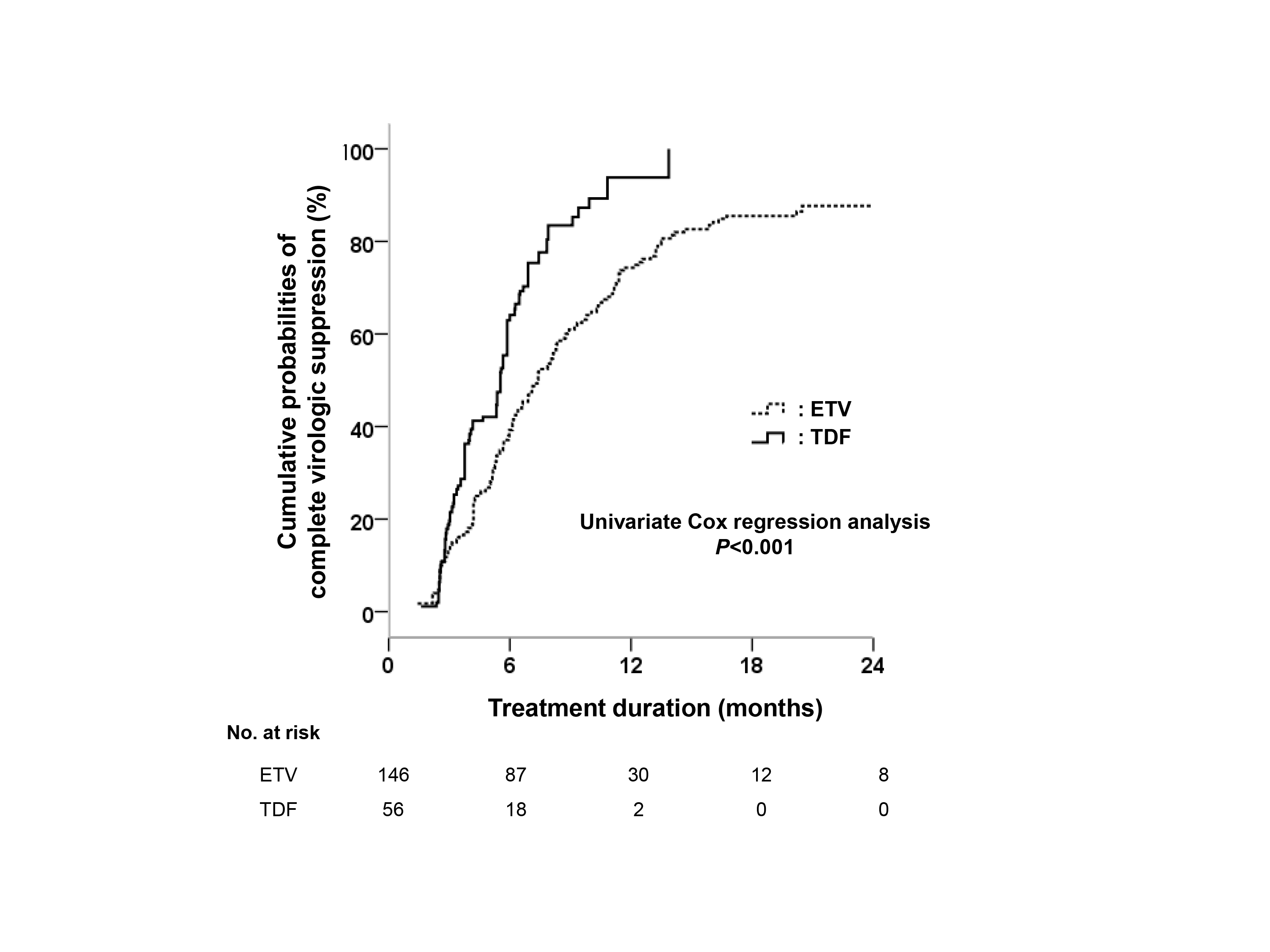

Supplement: S2 Fig — (TIF) [file pone.0130392.s002.tif]

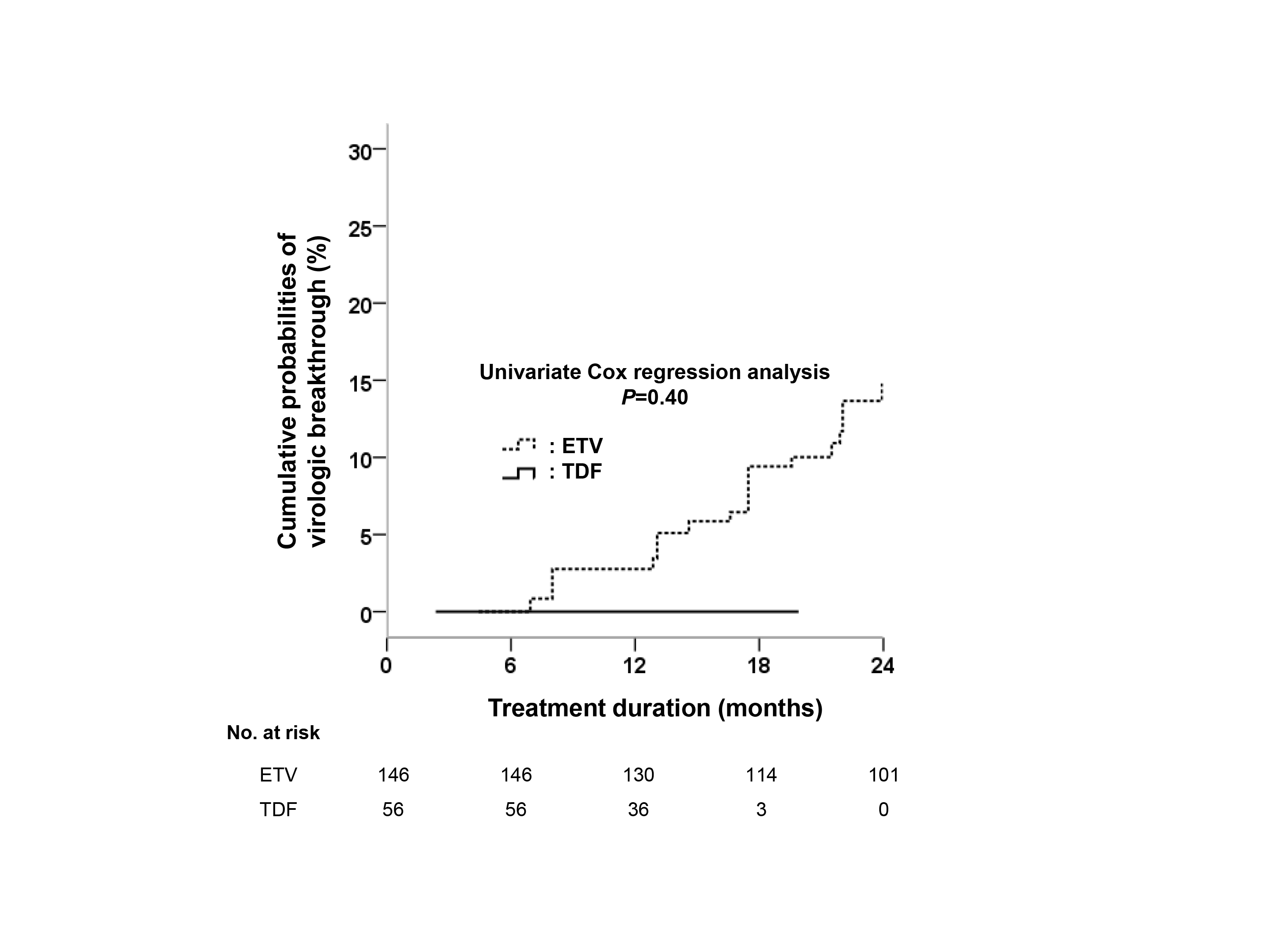

Supplement: S3 Fig — (TIF) [file pone.0130392.s003.tif]
