# Supplementary material for: Comparison of the Efficacy of Entecavir and Tenofovir in Nucleos(T)ide Analogue-Experienced Chronic Hepatitis B Patients
Source: PLoS One. 2015 Jun 29;10(6):e0130392. doi: 10.1371/journal.pone.0130392 (PMC4488001; doi:10.1371/journal.pone.0130392)
Supplement: S1 Table — (DOCX) [file pone.0130392.s004.docx]

**S1 Table. Summary of prior NA treatment regimens**

|  | ETV group (n=146) | TDF group (n=56) |
| --- | --- | --- |
| LAM/LdT/CLV monotherapy | 125 | 42 |
| Sequential LAM and LdT | 1 | 1 |
| Sequential LAM and CLV | 9 | 5 |
| Sequential LAM and ETV | 1 | 1 |
| Sequential LdT and ETV | 0 | 1 |
| Sequential LAM and ADV | 5 | 2 |
| Sequential CLV and ADV | 2 | 2 |
| Add–on combination with LAM and ADV | 3 | 2 |

ETV, entecavir; TDF, tenofovir disoproxil fumarate; LAM, lamivudine; LdT, telbivudine; CLV, clevudine; ADV, adefovir dipivoxil.
