# Supplementary material for: Comparison of the Efficacy of Entecavir and Tenofovir in Nucleos(T)ide Analogue-Experienced Chronic Hepatitis B Patients
Source: PLoS One. 2015 Jun 29;10(6):e0130392. doi: 10.1371/journal.pone.0130392 (PMC4488001; doi:10.1371/journal.pone.0130392)
Supplement: S2 Table — (DOCX) [file pone.0130392.s005.docx]

**S2 Table. Baseline characteristics by treatment group after inverse probability of treatment weighting**

|  | ETV group (n=146) | TDF group (n=56) | *P* |
| --- | --- | --- | --- |
| Age (years) | 50 (42-58) | 49 (42-58) | 0.79 |
| Male, n (%) | 82 (54.6) | 17 (46.1) | 0.35 |
| Baseline serum HBV DNA (log_10_ IU/mL) | 6.3 (4.4-7.5) | 6.2 (4.1-7.8) | 0.92 |
| Baseline serum ALT (IU/L) | 70 (35-172) | 92 (49-278) | 0.34 |
| Baseline serum creatinine (mg/dL) | 0.9 (0.8-1.0) | 0.8 (0.7-1.0) | 0.22 |
| HBeAg–positive, n (%) | 55 (36.7) | 15 (38.7) | 0.83 |
| Presence of cirrhosis, n (%) | 71 (47.2) | 15 (38.9) | 0.36 |
| Lines of prior treatment**^*^** | 1 (1-3) | 1 (1-3) | 0.67 |
| Duration of prior treatment (years) | 1.1 (0.7-2.1) | 1.5 (0.8-2.4) | 0.11 |
| CVS during prior treatment | 46 (30.6) | 10 (26.2) | 0.60 |
| Prior treatment with ADV, n (%) | 17 (11.4) | 3 (8.8) | 0.65 |

Unless otherwise indicated, data are medians, and data in parentheses are interquartile ranges. **^*^**Data are medians, and data in parentheses are ranges.

^†^Liver cirrhosis was diagnosed when the platelet count was below 100,000/mm3 and associated splenomegaly or esophageal-gastric varices were detected.

ETV, entecavir; TDF, tenofovir disoproxil fumarate; HBV, hepatitis B virus; ALT, alanine aminotransferase; HBeAg, hepatitis B e antigen; CVS, complete virological suppression; ADV, adefovir dipivoxil.
