# Supplementary material for: Comparison of the Efficacy of Entecavir and Tenofovir in Nucleos(T)ide Analogue-Experienced Chronic Hepatitis B Patients
Source: PLoS One. 2015 Jun 29;10(6):e0130392. doi: 10.1371/journal.pone.0130392 (PMC4488001; doi:10.1371/journal.pone.0130392)
Supplement: S3 Table — (DOCX) [file pone.0130392.s006.docx]

**S3 Table. Univariate and multivariate analyses of factors associated with complete virological suppression after inverse probability of treatment weighting**

|  | Univariate analysis | | Multivariate analysis | |
| --- | --- | --- | --- | --- |
| Variables | HR (95% CI) | *P* | HR (95% CI) | *P* |
| Age (per 10-year increase) | 1.017 (0.888–1.166) | 0.805 | - | 0.416 |
| Gender (male *vs.* female) | 0.928 (0.686–1.255) | 0.627 | - | 0.423 |
| Presence of cirrhosis | 1.238 (0.915–1.674) | 0.166 |  |  |
| HBeAg–positive | 1.992 (1.435–2.770) | < 0.001 | 0.545 (0.374–0.787) | 0.001 |
| Baseline serum HBV DNA (log_10_ IU/mL) | 0.891 (0.828–0.957) | 0.002 | 0.902 (0.831–0.980) | 0.015 |
| Baseline serum ALT (IU/L) | 1.000 (1.000–1.001) | 0.009 | 1.001 (1.000–1.001) | < 0.001 |
| Duration of previous treatment (year) | 0.979 (0.889–1.079) | 0.674 |  |  |
| Lines of prior treatment | 0.823 (0.586–1.155) | 0.259 |  |  |
| CVS during prior treatment | 1.776 (1.287–2.451) | < 0.001 | 1.406 (1.001–1.961) | 0.046 |
| Prior treatment with ADV | 1.204 (0.718-2.021) | 0.482 |  |  |
| Current regimen (TDF *vs.* ETV) | 1.976 (1.348–2.899) | < 0.001 | 2.141 (1.460–3.077) | < 0.001 |

HR, hazard ratio; CI, confidence interval; HBeAg, hepatitis B e antigen; HBV, hepatitis B virus; ALT, alanine aminotransferase; CVS, complete virological suppression; ADV, adefovir dipivoxil; ETV, entecavir; TDF, tenofovir disoproxil fumarate.
