# Supplementary material for: Comparison of the Efficacy of Entecavir and Tenofovir in Nucleos(T)ide Analogue-Experienced Chronic Hepatitis B Patients
Source: PLoS One. 2015 Jun 29;10(6):e0130392. doi: 10.1371/journal.pone.0130392 (PMC4488001; doi:10.1371/journal.pone.0130392)
Supplement: S4 Table — (DOCX) [file pone.0130392.s007.docx]

**S4 Table . Normalization of serum ALT**

| Outcome | ETV group | TDF group | *P* |
| --- | --- | --- | --- |
| Normalization of serum ALT | |  | 0.32^*^ |
| Baseline | 0/108 | 0/37 |  |
| Month 3 | 53/101 (52.5%) | 20/36 (55.6%) |  |
| Month 6 | 73/102 (71.6%) | 25/35 (71.4%) |  |
| Month 12 | 82/102 (80.4%) | 26/35 (74.3%) |  |
| Month 18 | 79/95 (83.2%) | 12/14 (85.7%) |  |

**P* value using generalized estimating equation analysis after adjustment for HBV DNA level, the presence of HBeAg, and history of CVS during prior treatment.

ETV, entecavir; TDF, tenofovir disoproxil fumarate; ALT, alanine aminotransferase; HBV, hepatitis B virus; HBeAg, hepatitis B e antigen; CVS, complete virological suppression.
