# Supplementary material for: Comparison of the Efficacy of Entecavir and Tenofovir in Nucleos(T)ide Analogue-Experienced Chronic Hepatitis B Patients
Source: PLoS One. 2015 Jun 29;10(6):e0130392. doi: 10.1371/journal.pone.0130392 (PMC4488001; doi:10.1371/journal.pone.0130392)
Supplement: S5 Table — (DOCX) [file pone.0130392.s008.docx]

**S5 Table. Univariate and multivariate analyses of factors associated with virological breakthrough after inverse probability of treatment weighting**

|  | Univariate analysis | | Multivariate analysis | |
| --- | --- | --- | --- | --- |
| Variables | HR (95% CI) | *P* | HR (95% CI) | *P* |
| Age (per 10-year increase) | 1.333 (0.952–1.867) | 0.094 | - | 0.813 |
| Gender (male *vs.* female) | 0.826 (0.411–1.660) | 0.592 | - | 0.587 |
| Presence of cirrhosis | 0.605 (0.294–1.244) | 0.172 |  |  |
| HBeAg–positive | 2.559 (1.262–5.191) | 0.009 | 2.746 (1.278–6.230) | 0.012 |
| Baseline HBV DNA (log_10_ IU/mL) | 1.128 (0.950–1.339) | 0.17 |  |  |
| Baseline serum ALT (IU/L) | 0.996 (0.992–0.999) | 0.025 | 0.994 (0.988–0.998) | 0.015 |
| Duration of previous treatment (year) | 1.149 (0.924–1.428) | 0.211 |  |  |
| Lines of prior treatment | 3.005 (1.539–5.867) | 0.001 | 3.605 (1.737–6.996) | < 0.001 |
| CVS during prior treatment | 0.044 (0.005–0.398) | 0.005 | 0.054 (0.002–0.301) | 0.011 |
| Prior treatment with ADV | 2.385 (0.844-6.742) | 0.101 |  |  |
| Current regimen (TDF *vs.* ETV) | 0.274 (0.002-2.188) | 0.400^*^ |  |  |

^*^by Firth’s correction.

HR, hazard ratio; CI, confidence interval; HBeAg, hepatitis B e antigen; HBV, hepatitis B virus; ALT, alanine aminotransferase; CVS, complete virological suppression; ADV, adefovir dipivoxil; ETV, entecavir; TDF, tenofovir disoproxil fumarate.
